# Parametric Appraisal of Process Parameters for Adhesion of Plasma Sprayed Nanostructured YSZ Coatings Using Taguchi Experimental Design

**DOI:** 10.1155/2013/527491

**Published:** 2013-10-28

**Authors:** Sisir Mantry, Barada K. Mishra, Madhusudan Chakraborty

**Affiliations:** ^1^CSIR-Institute of Minerals & Materials Technology, Bhubaneswar 751013, Odisha, India; ^2^Indian Institute of Technology Bhubaneswar, Bhubaneswar 751007, Odisha, India

## Abstract

This paper presents the application of the Taguchi experimental design in developing nanostructured yittria stabilized zirconia (YSZ) coatings by plasma spraying process. This paper depicts dependence of adhesion strength of as-sprayed nanostructured YSZ coatings on various process parameters, and effect of those process parameters on performance output has been studied using Taguchi's L_16_ orthogonal array design. Particle velocities prior to impacting the substrate, stand-off-distance, and particle temperature are found to be the most significant parameter affecting the bond strength. To achieve retention of nanostructure, molten state of nanoagglomerates (temperature and velocity) has been monitored using particle diagnostics tool. Maximum adhesion strength of 40.56 MPa has been experimentally found out by selecting optimum levels of selected factors. The enhanced bond strength of nano-YSZ coating may be attributed to higher interfacial toughness due to cracks being interrupted by adherent nanozones.

## 1. Introduction

Increased operating temperatures and hence improved performance of gas turbines or diesel engines can be realized by using thermal barrier coatings (TBC) [1–6]. Plasma sprayed thermal barrier coatings based on yttrium stabilized zirconia (YSZ) have been applied to hot section components. Zirconium based ceramics are considered to be best suitable for thermal barrier and wear resistance application due to its low density, high hardness, and low thermal conductivity. YSZ is the current industrial standard material of TBCs, owing to its low thermal conductivity, phase stability at relatively high temperatures, a relatively high coefficient of thermal expansion (CTE), and chemical inertness in combustion atmospheres as compared to other ceramics [7–11].

The advantages of plasma spraying include formation of ceramic microstructures with fine, equiaxed grains without columnar defects, deposition of graded coatings with a wide compositional variety, formation of thick coatings with only modest investment in capital equipment, and design capability for free standing thick forms of monolithic and mixed ceramics in near net shape configuration. Plasma spray coating is a typical thermal spraying process that combines particle melting, quenching, and consolidation in a single operation. It utilizes the exotic properties of the plasma medium to process different materials.

**Figure 1:**
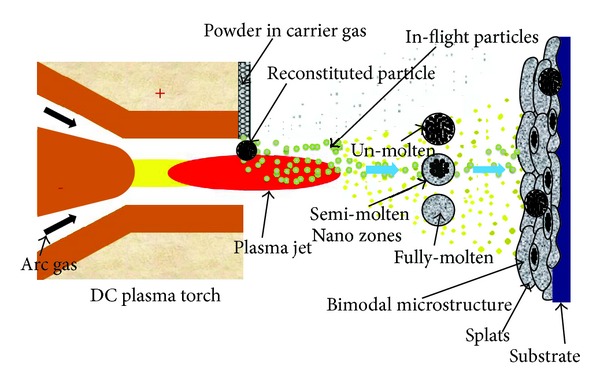
Schematic diagram of plasma spray coating process using nanostructured agglomerates.

YSZ coating has been proved to be more resistant against wear compared with other ceramic coatings. The higher wear resistance of the nanostructured coatings is attributed to their optimized microstructure and improved microhardness [12]. In recent years, there has been a growing interest in manufacturing and deposition of nanoscale powders. Bulk nanostructured material (grain size < 100 nm) has exhibited outstanding mechanical properties such as exceptional hardness, yield strength, and wear resistance [13–15]. Thermal spray coatings obtained from nanostructured powders (as shown in Figure 1) also exhibit such outstanding properties. Exceptional properties can be obtained if nanostructure of feedstock can be preserved during spraying and retained in the coating microstructure. Plasma spraying is a technique suited for this application because of short dwell time of powders at high temperature. However, process parameters must be carefully optimized to avoid grain coarsening and phase stability of materials [16].

Studies related to identification and quantification of phase transformations in plasma sprayed YSZ coatings have been done [17–19]. These characteristics are helpful in predicting the coating behavior under controllable plasma spray processes but are not sufficient in finding the means of a systematically optimal coating. In thermal barrier coating processes, appropriate use of modeling of a process model is rare. Detailed analysis of the relationship between independent variables and responses has not been established yet. Further, the impacts and importance of plasma spraying process factors on the surface coatings are still not well understood. The choice of parameters needs some understanding of the process as there are as many as 50 process variables [20]. To improve adhesion, all process parameters need to be understood, so as to undertake appropriate steps in the design of substrates and coating materials [21]. As the number of such process parameters is too large, statistical techniques could be employed for identification of significant process parameters for optimization. In this context, Taguchi experimental design offers an excellent tool for optimizing the total cost without compromising the performance output. In this work, this method has been adopted to investigate the influencing parameters like torch input power, molten particle velocity, stand-off distance, and powder feed rate on the adhesion strength of splat-substrate interface.

## 2. Experimental Details

### 2.1. Synthesis of Nanostructured Powders by Sol-Gel Route

The nanostructured YSZ powders employed in this study were synthesized through sol-gel technique [22]. A water based solution of zirconium oxychloride and yttrium oxide was prepared in order to get 8 mol% yttria stabilised zirconia (YSZ). Precursor materials were taken according to the proper stoichiometric, and an excess amount of nitric acid was added to the solution. Final solution was homogenized by constant stirring at 100–120°C temperature. After 5-6 h of constant stirring and heating, the translucent solution (with little amount of citric acid) was heated on a hot plate (at about 200 ± 250°C) until it turned into a black viscous gel, which on continued heating burned due to a vigorous exothermic reaction. Black ashes obtained after combustion were treated at 350°C in air for 1 h to eliminate the carbonaceous residues and calcined at 600°C for 2 h resulting in YSZ powder.

**Figure 2:**
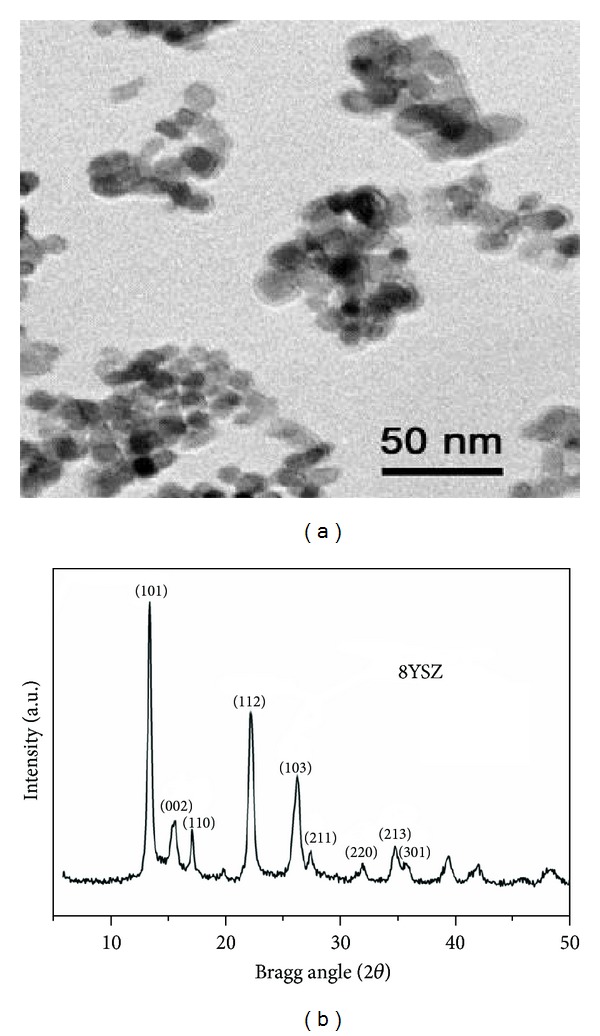
TEM micrograph and XRD pattern of as-synthesized YSZ particles.

XRD pattern of synthesized powder, as shown in Figure 2, reveals that the powder is composed of tetragonal zirconia phase (t-ZrO2). TEM image shows that the grain sizes of nanoparticles are between 20 and 30 nm.

### 2.2. Coating Material

**Figure 3:**
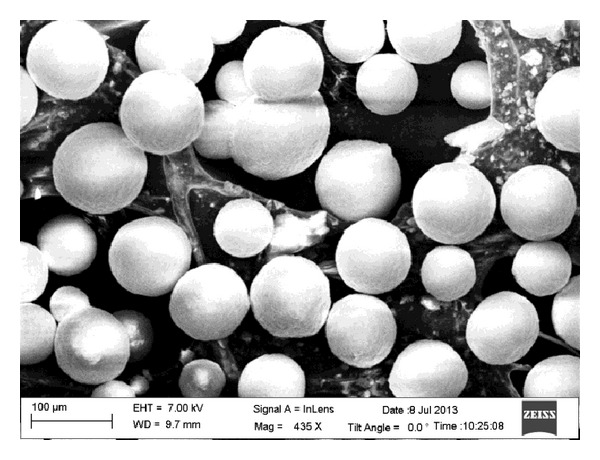
FESEM micrograph of spray dried YSZ particles.

The synthesized powders were then reconstituted to form micrometer-size agglomerates (40–70 *μ*m) that are large enough to be used as commercial powder feeders. The reconstitution process is done through spray drying [23]. The agglomeration of nano-YSZ was carried out in a Buchi B-290 research model spray dryer. A suspension of the nanoparticles was prepared using 300 mL of deionized H_2_O, 7 g of polyethylene glycol (PEG), and 70 g of nano-YSZ. The polymeric binder, PEG, in solution uses Vander Waals forces to bind the nanoparticles together and forms spherical droplets during atomization. The nano-YSZ slurry was attained via vigorous magnetic stirring for 30 min and heating to 300 K. The preheating of the solution assists in the slurry formation and in lowering the enthalpy needed during the drying process for moisture removal. The final product is a feedstock of size ~50 *μ*m spherical agglomerates containing nanograins of size 20–30 nm as shown in Figure 3.

## 3. Materials and Methods

The most important step in plasma spray coating technique is the preparation of the substrate surface in order to increase the mechanical anchoring between the substrate and the coating. The surface of the substrate was subjected to grit blasting to make the surface rough. In this grit blasting method, highly compressed air carrying alumina particles were bombarded on the surface to remove some material which made the surface rough. A uniform roughness of 6–8 *μ*m was maintained in order to provide better adhesion at the interface.

The coating process was carried out using an 80 kW plasma spray system supplied by M/s Metallization, UK. This is a typical atmospheric plasma spray system working in the nontransferred arc mode. The setup assembles a number of sub units like a plasma torch mounted on a six-axis robot, power supply (maximum power of 80 kW), powder feeders, mass flow controller, plasmagen gas supply, water chiller, and rotating turn table for sample rotation. The entire assembly is housed inside an acoustic chamber and is operated by a control console. In this study, high pure argon and helium were used as primary and secondary plasmagen gases, respectively, at an outlet pressure of 4 kg/cm^2^. A roughened Inconel 718 substrate of dimension 120 × 60 × 5 mm^3^ was fixed on the turn table, and YSZ agglomerates were sprayed at different torch input power levels. The process parameters are listed in Table 1. The number of passes was kept constant for each sample in order to make thickness of all coatings within similar range.

### 3.1. Adhesion Test

**Figure 4:**
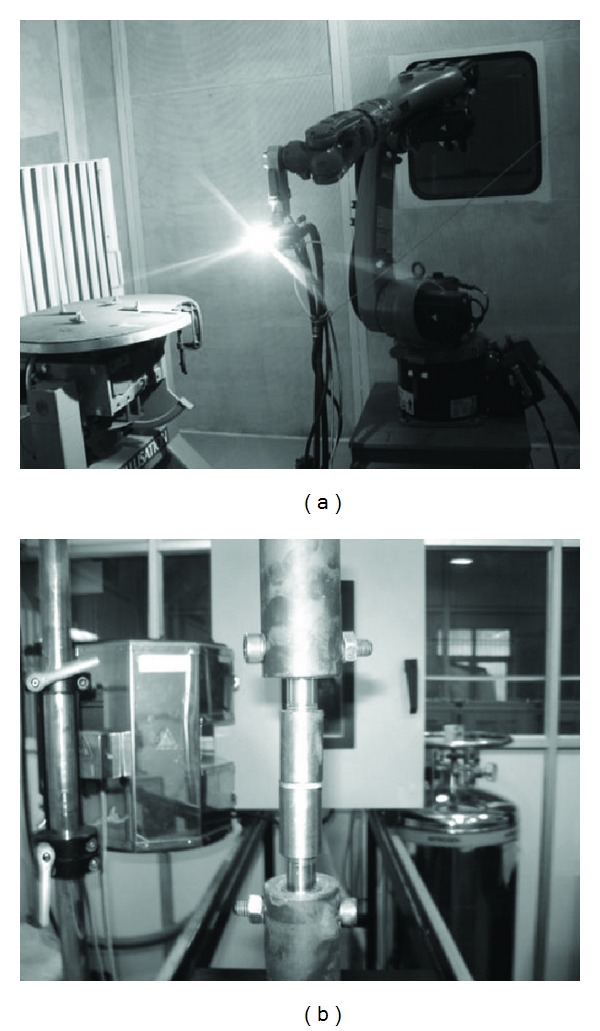
Plasma spraying torch and adhesion test setup as per ASTM C-633 standard.

To evaluate the coating adhesion strength, universal testing machine (make: INSTRON 8801) is used. The test is conducted by the pullout method as per ASTM C633 standard, as shown in Figure 4, in which two cylindrical specimens are taken. The face of one of the cylinders is coated by plasma spraying with the material under investigation. This coated face is glued with a resin HTK Ultra Bond 100 to the face of the other uncoated cylindrical specimen and kept in furnace at 150°C for approximately 1.5 hours for the setting of the glue. This uncoated face is to be sand blasted prior to the gluing. The assembly of the two cylinders is then subjected to gradual tensile load. The cross head speed was kept constant at 1 mm/min. The tensile strength, that is, the coating adhesion strength is calculated from the division of the maximum load applied at the rupture (i.e., failure occurs only at the coating-substrate interface) by the cross-sectional area of the cylindrical specimen considered.

Coating adherence tests have been carried out by many investigators with various coatings. However, it has been stated that the fracture mode is adhesive if it takes place at the coating substrate interface and that the measured adhesion value is the value of practical adhesion, which later is strictly an interface property, depending exclusively on the surface characteristics of the adhering phase and the substrate surface conditions. Taguchi experimental design is used to identify the most significant parameter affecting adhesion.

### 3.2. Online Particle Diagnostics

**Figure 5:**
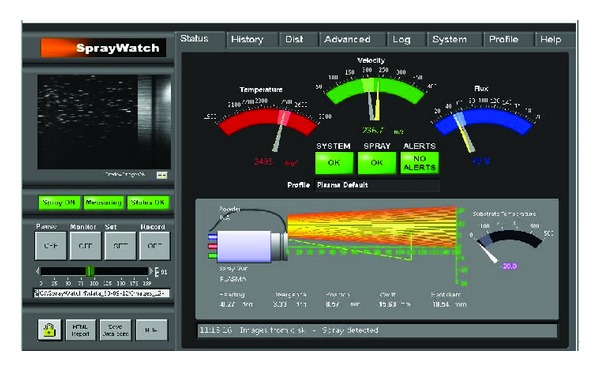
Particle velocity and temperature profile by CCD camera.

The coating properties are intimately linked to the properties of these lamellae, which in turn depend on inflight particle properties as well as substrate temperature during spraying. It is known that both performance and properties of coatings are influenced largely by the microstructure, which in turn is influenced by the particle state along with substrate and deposition conditions. Online diagnostics as shown in Figure 5 using Spray watch 2i equipment were carried out at different spray conditions to measure the particle velocity and temperature. Distance of the camera from the spray gun was equal to stand-off distance during coating manufacturing being 150 and 200 mm. Particle temperature determination is based on the two-color pyrometry, and in-flight particle velocities are measured from the length of the particle traces during known exposure times using a single high speed CCD camera [24].

### 3.3. Taguchi Experimental Design

In order to minimize effort and time required for testing without compromising the quality of a product or process, statistical methods are commonly followed [25]. Using these methods, the effect of every single condition in an experiment can be easily defined and studied. In the plasma spray coating process, the desired output, that is, the adhesion strength is dependent on many input factors. In order to study the influence of such factors on the performance output, the Taguchi experimental design is one of the best analysis tools. Using this design, the most significant factor out of large number of factors can be easily identified. It is a powerful analysis tool for modeling and analyzing the influence of control factors on performance output. The most important stage in the design of experiment lies in the selection of the control factors. Therefore, a large number of factors are initially included so that nonsignificant variables can be identified at the earliest opportunity. A literature review on the adhesion of plasma sprayed coatings revealed that parameters such as torch input power, particle velocity, powder feed rate, stand-off distance, and particle size largely influence the adhesion strength [26]. Splat formation depends on the impacting droplet velocity, size, molten state, impact angle, substrate roughness, and temperature [27]. 

The influence of these five factors with their four different levels on the adhesion strength of the coated sample is studied using L_16_ orthogonal array design. The control factors with their selected levels are shown in Table 2. The output result is further transforms into the signal-to-noise (S/N) ratios. Since the maximum adhesion is taken into consideration, S/N ratios are calculated for maximum under “larger is better” characteristics as a logarithmic transformation of loss function is given below.

“Larger the better” characteristic:

(1) $$\begin{array}{c} \frac{S}{N}=-10\log\frac{1}{n}\left(\sum \frac{1}{y^{2}}\right). \end{array}$$

## 4. Results and Discussion

Thickness of the nano-YSZ coatings on Inconel substrates is measured on the polished cross sections of the samples, using an optical microscope. Five readings are taken on each specimen, and the average value is reported as the mean coating thickness. The mean coating thickness is found to be 315 *μ*m. 

### 4.1. Surface Morphology

**Figure 6:**
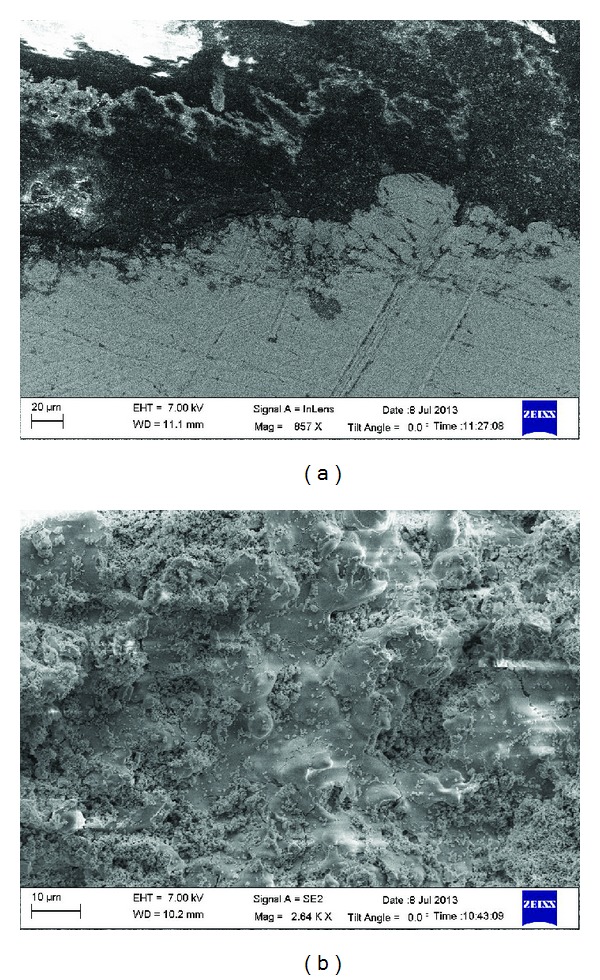
FESEM micrograph of as-sprayed nanostructured coating: (a) cross section, (b) surface.

Figure shows the surface morphology of the as-sprayed nanostructured YSZ coating obtained from field emission scanning electron microscopy (FESEM). Two different surface morphologies are observed. One is the dense and smooth zones, indicating good molten state of particles; the other one is the rough and porous zones, indicating unmolten or semimolten state of particles. The morphology of the cross section of the coating is shown in Figure 6. Splat boundaries of the molten and semimolten particles can be easily distinguished, which indicates that the powder was spheroidized during interaction with the plasma plume. After impingement on the substrate, molten particles form splats and solidify. The morphology of coatings reveals some regions of fully molten ceramic particles along with small pores. Particle distribution seemed to be uniform along the coating surface. 

### 4.2. Adhesion Analysis Using Taguchi Experimental Design

**Figure 7:**
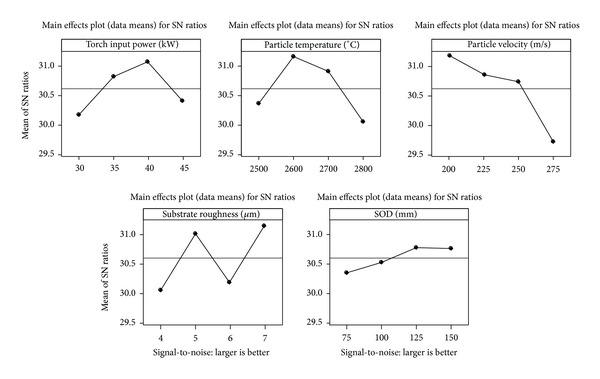
Main effect plot for S/N ratio of individual control factors.

The test results for the adhesion of the nano-YSZ coated substrate according to an L_16_ orthogonal design along with the corresponding S/N ratios are shown in Table 3. All five control factors are represented in second to sixth columns of the table, and the test results (i.e., adhesion strength) are presented in the seventh column. The adhesion test result for each run was the average of the experimental values obtained from three test runs. The S/N ratio for each test run was calculated and is shown in last column of Table 3. The overall mean value of the S/N ratios for the test run was 30.611 dB. The analysis was made using MINITAB14 software (CSIR-IMMT, Bhubaneswar, India), which is specifically used for design of experiment applications. The response table for the S/N ratio using the larger is better characteristics is shown in Table 4. In this table the delta value of the individual control factor based on the S/N ratio is shown, and a rank was accordingly assigned that indicates the significance of the control factors on the performance output. In this study, the molten particle velocity, with a higher delta value, was found to be the most significant factor, followed by the stand-off distance and particle temperature, influencing the adhesion of the interface of nano-YSZ coatings.  Figure 7 shows the main effect plot for S/N ratios of individual control factors. From the graphical analysis of this figure, it was concluded that maximum adhesion could be obtained with the combination of A3, B3, C1, D2, and E4, which is found to be 40.56 MPa. The higher bond strength achieved may be attributed to higher interfacial toughness due to cracks being interrupted by strong adherent nano-zones [28].

## 5. Conclusion

This experimental and analytical investigation on the nanostructured YSZ coated Inconel 718 substrate leads to the following important conclusions.

1. Successful coating deposition of as-synthesized nano-YSZ powder (by sol gel route) by plasma spraying route is possible on Inconel 718 substrates.
2. Retention of nanostructure is achieved by monitoring molten state of nanoagglomerates (temperature and velocity) by CCD camera and carefully optimizing process parameters.
3. From the Taguchi design method, particle velocity is found to be the most significant factor followed by SOD and particle temperature, influencing the adhesion strength of coated sample.
4. Maximum adhesion of 40.56 MPa is achieved experimentally by optimizing process parameters using Taguchi experimental design.

## Figures and Tables

**Table 1:** Selected operating parameters for plasma spray coating process.

Parameter	Operating range
Operating power	30–45 kW
Current	500–700 Amps.
Primary plasmagen gas (argon) flow rate	45–60 Lpm
Secondary plasmagen gas (helium) flow rate	5–15 Lpm
Nozzle to substrate distance (stand-off distance)	75–150 mm
Powder feed rate	25 gm/min

**Table 2:** Control factors with their selected levels.

	Levels				Unit
A: Torch input power	30	35	40	45	kW
B: Torch to base distance	75	100	125	150	mm
C: Molten particle velocity	200	225	250	275	m/sec
D: Particle temperature	2500	2600	2700	2800	°C
E: Surface roughness	4	5	6	7	*μ*m

**Table 3:** L_16_ Orthogonal array design with output and S/N ratio.

Test run	Torch input power (kW)	Particle temperature (°C)	Particle velocity (m/sec)	Substrate roughness (*μ*m)	SOD (mm)	Adhesion strength (MPa)	S/N ratio
1	30	2500	200	4	75	30.39	29.6546
2	30	2600	225	5	100	36.67	31.2862
3	30	2700	250	6	125	32.89	30.3413
4	30	2800	275	7	150	29.45	29.3817
5	35	2500	275	6	100	28.72	29.1637
6	35	2600	250	7	75	38.75	31.7654
7	35	2700	225	4	150	35.29	30.9530
8	35	2800	200	5	125	37.12	31.3922
9	40	2500	225	7	125	38.71	31.7565
10	40	2600	200	6	150	39.37	31.9033
11	40	2700	275	5	75	33.90	30.6040
12	40	2800	250	4	100	31.59	29.9910
13	45	2500	250	5	150	34.79	30.8291
14	45	2600	275	4	125	30.43	29.6660
15	45	2700	200	7	100	38.49	31.7070
16	45	2800	225	6	75	29.50	29.3964

**Table 4:** Response table for signal-to-noise ratios.

Level	A	B	C	D	E
1	30.17	30.35	31.16	30.07	30.36
2	30.82	31.16	30.85	31.03	30.54
3	31.06	30.90	30.73	30.20	30.79
4	30.40	30.04	29.70	31.15	30.77
Delta	0.90	1.11	1.46	1.09	0.43
Rank	**4**	**2**	**1**	**3**	**5**
